# Differential Diagnosis of Pediatric Multiple Sclerosis

**DOI:** 10.3390/children6060075

**Published:** 2019-06-03

**Authors:** Maria Milagros Galardi, Cristina Gaudioso, Saumel Ahmadi, Emily Evans, Laura Gilbert, Soe Mar

**Affiliations:** Department of Neurology, Washington University in St. Louis, St. Louis, MO 63110, USA; mmgalardi@wustl.edu (M.M.G.); gaudioso@wustl.edu (C.G.); saumel@wustl.edu (S.A.); eevans23@wustl.edu (E.E.); l.gilbert@wustl.edu (L.G.)

**Keywords:** demyelination, pediatric multiple sclerosis, NMOSD, MOG-ab, ADEM, leukodystrophies, metabolic disorders

## Abstract

The differential diagnosis of pediatric multiple sclerosis (MS) can be broad and pose diagnostic challenges, particularly at initial presentation. Among demyelinating entities, neuromyelitis optica spectrum disorders (NMOSD), myelin oligodendrocyte glycoprotein antibodies (MOG-ab) associated disorders, and acute disseminated encephalomyelitis (ADEM) are now well-known as unique disease processes and yet continue to overlap with MS in regards to clinical presentation and imaging. In non-inflammatory entities, such as metabolic disorders and leukodystrophies, an erroneous diagnosis of MS can be made even while applying appropriate diagnostic criteria. Knowing the epidemiology, typical clinical presentation, diagnostic criteria, and ancillary test results in each disease, can aid in making the correct diagnosis by contrasting these features with those of pediatric MS. Determining the correct diagnosis early, allows for efficient and effective treatment as well as appropriate prognostication.

## 1. Introduction

Differentiating pediatric multiple sclerosis (MS) from a wide range of disorders of both inflammatory and non-inflammatory etiologies that present in a strikingly similar way, remains a challenge in our daily clinical practice. When a child with acute neurologic symptoms is found to have white matter abnormalities, there are a variety of factors that should be taken into consideration in the pursuit of the most likely diagnosis. These include epidemiologic data, presenting signs and symptoms, diagnostic criteria, and ancillary tests. Specifically, imaging patterns and laboratory testing results ranging from the broader cerebrospinal fluid (CSF) cell and protein profiles to more specific antibodies in both blood and CSF can aid in differentiating pediatric MS from other disorders. The importance of establishing the correct diagnosis early has significant implications in selecting the most optimal treatment. With advances in research, we now know of options that may be effective for pediatric MS, but are ineffective and sometimes even detrimental in other disease processes. Our review focuses on differential diagnoses that are commonly mistaken for MS, as we attempt to compile what the most recent literature defines in terms of epidemiology/pathophysiology, clinical presentation, diagnosis, and treatment/prognosis for each. As a starting point for each disease or disease category, we describe a clinical case seen at our own institution that delineates the challenge of differentiating these entities from pediatric MS.

## 2. Neuromyelitis Optica Spectrum Disorders

### 2.1. Clinical Case

**Figure 1:**
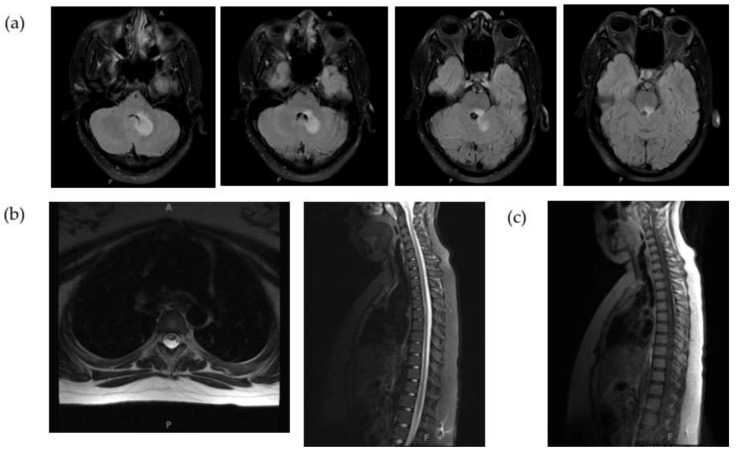
Brain and spine MRI of patient at initial demyelinating event. (**a**) T2 hyperintense lesion predominantly located within the white matter of the left cerebellar hemisphere extending to the brachium pontis and posterior brainstem. (**b**) Intramedullary T2 hyperintense lesion along the right aspect of the cord at T4–T5. (**c**) Mild enhancement of spine lesion.

A thirteen-year-old girl presented with several days of right-sided torticollis, gaze impairment, left-sided weakness, and changes in speech. Neurologic exam was notable for pseudobulbar affect, intranuclear ophthalmoplegia, left hemiparesis, and ataxia. Brain magnetic resonance imaging (MRI) showed a T2 hyperintense white matter lesion on left cerebellar hemisphere extending to the brainstem, with associated restricted diffusion and mild peripheral enhancement (Figure 1a). A spine MRI showed an intramedullary T2 hyperintense lesion of the cord at T4–T5 with mild enhancement (Figure 1b,c). Visual evoked potentials revealed reduced amplitudes bilaterally. A lumbar puncture showed 123 nucleated cells with lymphocytic predominance, normal protein and IgG index, and no oligoclonal bands. Serum NMO immunoglobulin G (IgG) was negative. The patient was initially diagnosed with clinically isolated syndrome (CIS) with brain stem and cerebellar presentation, with high risk for MS. She was treated with a five-day course of high dose steroids, followed by two doses of intravenous immunoglobulin (IVIG) and inpatient rehabilitation because of slow and poor recovery. She recovered significantly and was ambulatory at the time of discharge. Two months after initial presentation, she was readmitted with recurrence of gait instability, slurred speech, and left-sided weakness. Repeat brain MRI showed interval progression of the demyelinating process now involving the superior vermis and middle cerebellar peduncle with new patchy enhancement as well as longitudinal transverse T4–T7 T2/stir hyperintensity with cord swelling and patchy contrast enhancement. Lumbar puncture showed 13 nucleated cells, normal protein, high IgG index and six oligoclonal bands (one in serum). Aquaporin 4 antibodies (AQP4-ab) were positive and she was diagnosed with neuromyelitis optica (NMO).

### 2.2. Epidemiology and Pathophysiology

NMOSD are central nervous system (CNS) demyelinating conditions which primarily affect the optic nerves and spinal cord via unique pathophysiologic mechanisms and are different from the classic CNS demyelinating condition of MS. Pediatric NMOSD accounts for about 4% of total NMO cases in the United States [1]. Disease onset occurs at about 10 years of age, which is similar to MS (13 years), but higher than ADEM (5 years). Disease onset before 11 years of age is more common in ADEM (96%) than MS (20%) and NMO (54%) [1]. Among children younger than 11 years of age at disease onset, the female to male ratio in MS has been reported to be 1.1:1, while NMO is more common in females (1.5:1). These gender differences are further accentuated in patients ≥11 years of age, with MS and NMO being more common in females (F:M of 1.86:1 for MS, 3.25:1 for NMO) [1]. This highlights the effect of sex hormones on the onset of these demyelinating conditions, which is supported by the fact that pregnancy affects disease severity for NMO and MS [2,3,4]. NMOSD epidemiology also displays racial variation. Asian and Afro-American/Afro-European populations have a younger mean age of onset than Caucasians, and Afro-American/Afro-European populations are more likely to have a severe attack at onset than Asian and Caucasian populations [5].

The discovery of the NMO-IgG antibody which was found to target the aquaporin-4 water channel (AQP4), did not only provide a reliable biomarker for the diagnosis of NMO, but also helped in the understanding of the disease process [6,7,8]. Specifically, AQP4 loss is seen in astrocytes, and NMO has been proposed to be an immune astrocytopathy, driven mainly by antibodies against AQP4, and hence also referred to as an immune aquaporinopathy [9,10,11]. It is primarily considered to be a humoral immune-driven pathologic process, whereas MS was classically considered to be a cell-mediated pathologic process. However, we now know that MS can involve both humoral and cell-mediated arms of the immune system attacking the CNS, and hence therapies targeting T cells, B cells or antibodies produced by plasma cells, can work in MS, depending on the major pathologic process involved in individual MS patients [12]. This is in contrast to NMOSD, where B cell and antibody clearance strategies are effective, but certain MS therapies that do not target humoral immunity can exacerbate disease [11,13,14,15,16].

NMO also involves the sensitization of Th17 cells to AQP4 peptides. These Th17 cells help the B cells that are activated by conformationally intact AQP4 proteins, thereby producing AQP4-ab [11]. Hence, T cells do not directly cause astrocyte damage in NMO, but help potentiate the humoral response, and this immunologic process is driven outside the CNS [17]. NMO pathogenesis likely involves both a genetic and an environmental component. From the genetic standpoint, NMOSD seems to be associated with human leukocyte antigen HLA-DRB1*03, which is also associated with other autoimmune conditions like systemic lupus erythematous (SLE) [18]. This is in contrast to MS, which is associated with HLA-DRB1*1501, and this variant is not associated with NMOSD [18]. Environmentally, NMO could be related to gut dysbiosis, as it has been suggested that patients with NMO have an overabundance of Clostridium perfringens in their gut [19]. From the molecular standpoint, the ABC transporter permease of C. perfringens has a peptide sequence which shares 90% homology to a region of the AQP4 protein, which can explain cross-reactivity between these proteins [20].

### 2.3. Clinical Presentation

As encompassed in the term “neuromyelitis optica”, the typical clinical presentation of NMOSD is that of optic neuritis (ON) and/or longitudinally extensive transverse myelitis (LETM) (defined as lesions spanning >3 complete vertebral segments), in the context of seropositivity for AQP4-IgG antibodies [6,21]. Clinically, NMO (previously referred to as Devic’s disease) was first described by Eugène Devic and Fernand Gault in 1894 at Congrès Français de Médecine in Lyon, France [22]. The distinction between NMO and MS has been evolving ever since, taking into account not only the differences in clinical presentation but also in imaging and serology.

The most common presenting symptoms in the pediatric population include visual and motor impairment, seizures, and constitutional symptoms like fever and vomiting [1]. The most common localizations for a primary presentation are to optic nerve, brainstem, and spinal cord [1]. The area postrema can be selectively affected in NMO, and vomiting has been reported as a presenting symptom in 38% of pediatric NMO patients [1,23]. It should be noted that the majority of children with AQP-4 seropositive NMO have at least one episode of ON (83%) or LETM (78%) [24].

### 2.4. Diagnosis

The discovery of AQP4-IgG revolutionized the diagnosis of NMOSD, providing an objective and reproducible tool for diagnosing this entity and differentiating it from MS [6,25]. However, NMOSD can be diagnosed in the absence of AQP4-IgG antibodies if additional criteria are met [25]. The clinical case above highlights the importance of having NMOSD on the differential when the complete diagnostic criteria might not be met initially at presentation, as the disease can be seronegative for AQP4-IgG. Although relapsing disease is more common in the pediatric population, it should be noted that monophasic NMO tends to be seronegative for AQP4-IgG when compared to relapsing disease [26].

**Figure 2:**
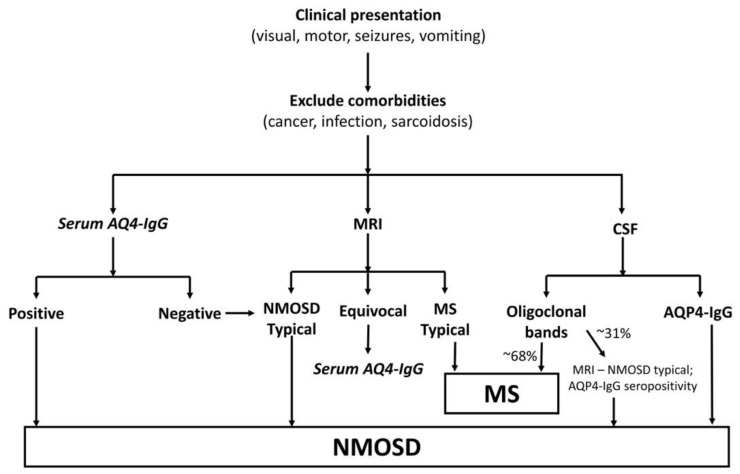
Schematic aid for the diagnosis of neuromyelitis optica spectrum disorders (NMOSD) (Modified from Wingerchuk et al., 2015) [25]. As noted in this review, NMOSD should be on the differential when the clinical presentation involves optic nerve, brainstem, or spinal cord. It would be important to rule out other causes of focal findings, which include many of the comorbidities listed. Serology and MRI are the highest yield tests to reach a definitive diagnosis of NMOSD, and CSF analysis is usually warranted when the diagnosis is ambiguous or other pathologies are suspected. If MRI findings are equivocal, it would be appropriate to rely on serology and cerebrospinal fluid (CSF) analysis. Presence of oligoclonal bands (OCBs) cannot faithfully differentiate between multiple sclerosis (MS) and NMOSD but would suggest MS (~68% cases) diagnosis more than NMOSD (~31% cases), unless MRI/serology definitively indicate NMOSD [1,25].

The US network of pediatric MS centers report showed that 97% of pediatric NMO patients met the revised NMOSD 2015 criteria as defined by the international panel on NMO diagnosis (IPND). This was a remarkable improvement from the 2006 Wingerchuk criteria met by only 49% pediatric NMO patients [21]. The new criteria take into account patients who are seronegative for AQP4-IgG antibody or where such testing is not available, by making clinical and imaging requirements more stringent (Figure 2). It defines six core clinical characteristics including: ON, acute myelitis, area postrema syndrome (hiccups, nausea or vomiting), acute brainstem syndrome, acute diencephalic syndrome (or symptomatic narcolepsy), and symptomatic cerebral syndrome. In the absence of AQP4-IgG, it is still possible to diagnose NMOSD with more stringent clinical and radiologic criteria: presents with at least two different core clinical characteristics, one of which is either ON, acute myelitis with LETM, or area postrema syndrome, and must also fulfill additional MRI criteria characteristic of NMOSD.

The IPND 2015 took into account the recommendations from the Pediatric Working Group members and found the new diagnostic criteria to be compatible for pediatric patients, with a few caveats: acute myelitis associated with LETM lesion on MRI may be less specific in pediatric NMOSD, and more children present with monophasic LETM [25]. The decrease in specificity (increase in false positivity) of LETM MRI lesions in the pediatric population is proposed to be due to the presence of LETM lesions in pediatric MS (~15%) and pediatric monophasic ADEM. Additionally, monophasic LETM in the pediatric population is usually seronegative for AQP4-IgG [27]. This highlights the fact that LETM lesions in isolation are not as predictive of NMOSD in the pediatric population [25,27]. Moreover, about 45% of children who are seropositive for AQP4-IgG, experience recurrent cerebral manifestations including encephalopathy. Hence, IPND 2015 proposes that children who present with polyfocal demyelinating lesions with encephalopathy and have seropositivity for AQP4-IgG, should have a diagnosis of NMOSD over ADEM [25].

From an imaging standpoint, MRI is the most widely used and accepted modality to aid in the diagnosis of NMOSD, and differentiates it from MS and other demyelinating conditions. Characteristic MRI findings suggestive of NMOSD include optic neuritis lesions extending greater than ½ optic nerve length, involvement of the optic chiasm, extensive intramedullary myelitis lesions involving >3 spinal segments, and diencephalic lesions [25]. Other rare but characteristic MRI findings for NMOSD include the involvement of the area postrema in the dorsal medulla and periependymal brainstem lesions [25]. T2 lesions involving <3 complete vertebral segments, predominantly peripheral cord lesions (>70%), are suggestive of MS over NMOSD [25].

In pediatric NMOSD, CSF collected during an acute ON episode can be bland, while during a myelitis episode CSF may be more indicative of active inflammation [28]. During the remission phase of NMOSD, CSF can be less inflamed than during attack onset or relapse [29]. In an acute attack of NMO, CSF typically displays significant pleocytosis with >100 nucleated cells, with a predominance of neutrophils or lymphocytes [1,28]. This is in contrast to MS, where pleocytosis is rarely seen and is predominantly lymphocytic if present [1]. Oligoclonal bands (OCBs) which are classically seen in MS, are present in ~31% of pediatric NMOSD patients (versus ~68% pediatric MS patients) [1,30]. The decreased frequency of CSF restricted OCBs in NMOSD has been thought to be due to the absence of intrathecal IgG synthesis in NMO [31]. This is also supported by the fact that IgG index is elevated in only ~30% of pediatric NMO patients vs. 63% of MS [1]. The elevation of CSF protein is higher in NMOSD than MS, correlating with the fact that NMO is a disease process beginning at the astrocyte foot processes, thereby leading to a leaky blood–brain barrier (BBB) [31]. NMO specific CSF AQP4-IgG testing can be done in suspected NMOSD cases, especially in those who are seronegative for AQP4-IgG [28]. However, it is rare to have AQP4-IgG in CSF and not serum [32,33].

Interestingly, the term opticospinal MS originated in Japan and has been used to describe a specific pattern of MS, which is more prevalent in Asian countries [25,34]. It was an important milestone because it described a distinct form of MS which primarily affected the optic nerve and spinal cord. It is controversial whether opticospinal MS is one of the NMO spectrum disorders which includes AQP-4 IgG and MOG seropositivity, or if it is a separate entity, especially in patients who are seronegative for both AQP-4 and MOG antibodies. The IPND 2015 guidelines concluded that opticospinal MS should be considered an NMO spectrum disorder [25].

Generally speaking, diagnostic features suggestive of MS over NMOSD include a progressive overall clinical course, presence of partial transverse myelitis not associated with LETM MRI lesions, and presence of typical MS MRI findings [25].

### 2.5. Treatment and Prognosis

As a standard of clinical measure, the Extended Disability Scoring System (EDSS) has been used to monitor changes in the overall functioning of patients with MS and NMOSD [35]. Over a period of two years after onset in the pediatric population, the number of attacks and EDSS scores were higher in patients with NMO when compared to MS [1].

With regards to treatment, it is crucial to distinguish NMOSD from MS early on in the disease process, since certain therapies for MS like β-interferon [13], fingolimod [15], natalizumab [16], alemtuzumab [14], can aggravate NMO and even increase AQP-4 IgG titers [13,36].

If untreated, acute exacerbations of NMOSD are more severe than MS exacerbations and the recovery is minimal [1,37]. Hence, it is important to treat acute exacerbations and start suppressive therapy that should be continued during disease remission. Every NMOSD attack leads to cumulative neurological damage and disability [37,38]. In the pediatric population, 93–95% have relapsing disease [1,24]. Since acute events are primarily inflammatory in nature, the goal is to suppress this inflammatory attack, which can be achieved with steroids. Plasma exchange (PLEX) is also used in NMO as a standalone therapy or in conjunction with steroids. There is good evidence in the adult population that PLEX in conjunction with IV methylprednisolone (IVMP) is better than steroids alone [39,40].

With regards to suppressive immunotherapy, the most commonly used agents are Rituximab, azathioprine, and mycophenolate mofetil (MMF) [41,42,43,44]. Rituximab or MMF are preferred over azathioprine [1]. Benefits of using Rituximab are a faster onset of action [41] and the fact that its tolerability and dosing have been verified in the pediatric population [42]. If the diagnosis of NMOSD vs. MS is unclear, it would be appropriate to use NMOSD suitable immunotherapy because of the risk of NMOSD exacerbation with certain MS therapies [11].

Recently, targeting of the complement system arm of the humoral immunity has been shown to be beneficial in the adult population by repurposing of the terminal complement inhibitor eculizumab in NMOSD [45]. This is a promising avenue with regards to management of NMOSD, but currently, the use of this in the pediatric population is limited given the lack of data and its cost.

Pediatric NMOSD patients can have a longer time to disability compared to adult-onset NMOSD with most pediatric patients surviving into adulthood [46]. Mortality rates for adult NMOSD range between 9 and 32% worldwide over 55.2 to 75 months from diagnosis [5,47,48]. This is driven by lesions involving the brainstem [49]. With the advent of suppressive immunotherapy, disease morbidity and mortality should decline. Since NMOSD is rarely secondarily progressive, maintaining attack-free intervals with immune therapies is highly beneficial from a neurological stability standpoint [11,37]. In the future, the hope is to have personalized therapies for NMO. There is ongoing work in this direction, as demonstrated by the use of FCGR3A polymorphisms in predicting response to Rituximab as well as the development of therapies directly targeting AQP-4 IgG binding to AQP-4 [50,51,52].

## 3. MOG Antibody-Associated Disorders

### 3.1. Clinical Case

**Figure 3:**
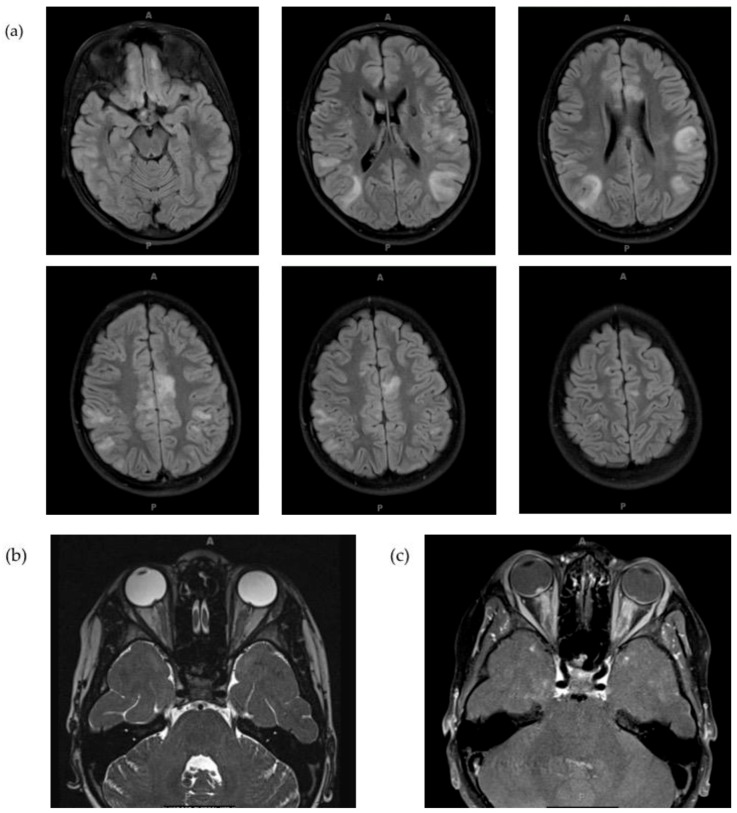
Brain MRI of patient at initial demyelinating event. (**a**) Multiple areas of large diffuse cortical and juxtacortical T2/FLAIR hyperintensities involving bilateral frontal, temporal, parietal and occipital areas. (**b**)Thickening of the optic nerves with abnormal T2 signal of the bilateral intraorbital, canalicular and prechiasmatic optic nerves with evidence of bulging of the optic disc and flattening of the posterior sclera. (**c**) Contrast enhancement.

An eight-year-old boy presented with acute onset, painful, bilateral vision loss. Neurologic exam was only notable for impaired visual acuity. Initial work-up included brain MRI showing thickening of the optic nerves and multiple areas of cortical and juxtacortical T2/FLAIR hyperintensity involving the bilateral frontal, temporal, parietal, and occipital lobes, as well as the corpus callosum, with contrast enhancement of some lesions (Figure 3). These findings fulfilled a diagnosis of pediatric MS [53]. Spine MRI was normal. A lumbar puncture showed 32 nucleated cells, normal protein, IgG index, no oligoclonal bands, AQP4-ab negative. The patient was treated with five days of high-dose IV steroids, followed by a three-week oral taper. His vision improved to almost normal. He had additional episodes of optic neuritis with negative workup for infection and rheumatologic disorders except for MOG antibody as it was not available at that time. He was diagnosed with MS, treated with glatiramer acetate with initial resolution of brain lesion, followed by multiple clinical relapses, resulting in a change in his disease-modifying therapy to natalizumab. He continued to have clinical relapses with interval progression of brain MRI lesions, which prompted the referral to us. By that time, testing for MOG-ab had become clinically available, was obtained, and resulted positive. The patient was ultimately diagnosed with recurrent optic neuritis associated with MOG antibody seropositivity. Natalizumab was discontinued and IVIG was started.

### 3.2. Epidemiology and Pathophysiology

Myelin oligodendrocyte glycoprotein antibodies (MOG-ab) have been identified in a range of acquired demyelinating syndromes in the pediatric population, including acute demyelinating encephalomyelitis (ADEM), optic neuritis (ON), and transverse myelitis (TM) [54,55,56,57]. When comparing between MOG seropositive and seronegative pediatric patients with acute demyelinating diseases, MOG seropositive patients are significantly younger at the disease onset than seronegative patients. The groups do not differ significantly in terms of gender, race, or ethnicity [58,59]. Studies have also suggested age-dependent phenotypes among MOG-ab seropositive pediatric patients. Specifically, encephalopathy is more common in younger patients (four to eight or nine years of age), while optic neuritis is more common in older patients (13 to 18 years) [58,60].

MOG is a CNS myelin-specific glycoprotein. While it is quantitatively a minor component of myelin (0.05%), its location on the outermost surface of the myelin sheath and in the membrane of oligodendrocytes [61,62] makes it accessible to antibodies and a target for auto-antibody mediated diseases. MOG was first identified as a target of demyelinating antibodies in animal models and in experimental autoimmune encephalomyelitis (EAE) [63,64]. Several subsequent studies have demonstrated its role as an autoantigen for T and B cell responses [65] and have shown that MOG-ab are capable of inducing complement-mediated cytotoxicity and augmenting demyelination [55,57]. In children, the anti-MOG humoral immune response has been shown to be specific for demyelinating CNS diseases and can differentiate them from viral encephalitis [66]. While it remains unclear if MOG-ab have demyelinating activity, the data suggests they do not merely reflect myelin destruction [59].

### 3.3. Clinical Presentation

MOG-ab have been identified in both monophasic and polyphasic forms of demyelinating CNS diseases. Transient MOG-ab have been associated with a monophasic disease course, whereas persisting MOG-ab have been associated with a recurrent disease course [56,67,68,69]. In a study looking at 210 children with acquired demyelinating syndromes, 22/65 (33.8%) of MOG-ab–positive children experienced a clinical relapse. These children tended to have persistently high MOG-ab titers (after 24 months). They were additionally characterized by higher age at onset (median age 8 vs. 4.5 years), female sex predominance, and optic nerve involvement [70]. When compared to other clinical presentations of pediatric demyelinating disease in another study looking at 117 children with acute demyelinating disease, the group of children with polyfocal-disease onset plus encephalopathy (meeting the IPMSSG criteria for ADEM) had the highest frequency of anti-MOG seropositivity (42%). Additionally, 18% of children presenting with polyfocal acute demyelinating disease without encephalopathy had MOG antibodies. [59]. Recent studies have suggested that MOG-ab are almost exclusively associated with non-MS disease courses [54,59,70]. Furthermore, it has been proposed that even if these antibodies are present in MS patients, their titers are lower, in comparison to ADEM patients [59,68,71].

### 3.4. Diagnosis

Several authors agree on pleocytosis and rare oligoclonal bands (OCB) being classic findings in the CSF of children with MOG-ab associated demyelinating disease [60,67,70,72]. Pleocytosis is also common in pediatric MS, but OCB have been reported in over 90% of patients on presentation [73].

Studies have reported the prevalence of MOG-ab to be 31–42% among patients with childhood acute demyelinating syndromes [54,70,74]. The reported prevalence of MOG-ab among pediatric patients with NMOSD specifically, has been much more variable ranging from 14 to 80% in AQP4 seronegative patients [56,58,59,74,75]. Among these studies, the prevalence of MOG-ab in AQP4 seropositive patients was 0%.

From an imaging standpoint, the MRI burden of lesions in the brain and spine has been shown to be extensive in patients with MOG-ab-positive pediatric demyelinating diseases. Thulasirajah et al. noted confluent and asymmetrical subcortical and deep white matter lesions, as well as longitudinally extensive spinal cord lesions [72]. Similarly, Bauman et al. found that the brain MRI of children with ADEM and MOG-ab was characterized by large, hazy, bilateral widespread lesions without clear boundaries. They also observed an increased frequency of longitudinal extensive transverse myelitis (LETM) [67]. Moreover, the presence of corpus callosum (CC) lesions has been described to be a statistically significant difference between MOG seropositive and seronegative patients, with a retrospective study showing that CC lesions were present in 52% of seronegative patients and 0% of the seropositive patients [58].

The development of cell-based assays (CBA) using transfected cells has impacted how anti-MOG antibodies are measured and has facilitated the identification of clinically relevant MOG-IgG. Prior detection methods, based on western blotting or ELISA, were less accurate due to their inability to distinguish specific antibodies against conformational MOG epitopes, specifically those that have been shown to be biologically significant [76]. CBA offers an advantage in this sense but still poses the issue of technical heterogeneity, leading to sometimes variable results [77]. Overall, a titer of ≥1:160 has been used to indicate MOG-ab-positivity by several authors [54,70,72,78].

### 3.5. Treatment and Prognosis

It appears that children with anti-MOG-ab demyelinating syndromes have rapid resolution of both clinical and imaging findings following steroid and IVIG therapy [72].Children with NMOSD with positive anti-MOG-IgG antibodies may have a more benign disease course (than those with anti-AQP4 antibodies) that is responsive to milder forms of immunomodulation [69]. Although disease-modifying drugs are extensively used in the treatment of patients with MS, a recent prospective study showed that they are not associated with clinical improvement in patients with relapsing MOG-ab associated disease. Rather, intravenous immunoglobulins (IVIG), rituximab, mycophenolate mofetil, and azathioprine (in descending order of efficacy) have been associated with a reduction in relapse frequency [60].

## 4. Acute Disseminated Encephalomyelitis

### 4.1. Clinical Case

**Figure 4:**
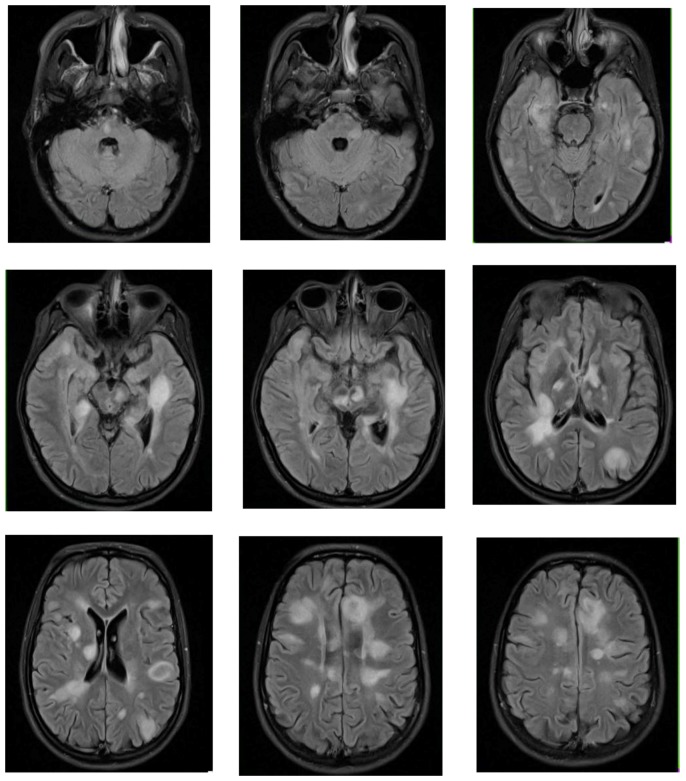
Brain MRI of patient at initial demyelinating event demonstrating numerous T2 hyperintense ovoid shaped lesions scattered throughout the periventricular and subcortical white matter of the cerebral hemispheres, as well as of the corpus callosum, cerebral peduncles, midbrain, brachium pontis, and pons.

A seventeen-year-old girl presented with five weeks of progressive confusion, encephalopathy, slurred speech, gait instability, and significant weight loss. Neurologic exam was notable for altered mental status with the ability to follow simple commands, but no speech output, appendicular hypertonia, diffuse hyperreflexia, and gait described as shuffling. Initial brain MRI showed numerous T2 hyperintense, T1 hypointense ovoid shaped lesions scattered throughout the periventricular and subcortical white matter of the cerebral hemispheres, as well as of the corpus callosum, cerebellum, and brain stem with some enhancing lesions (Figure 4). CSF was obtained and showed no nucleated cells, normal protein, no oligoclonal bands, and elevated myelin basic protein. She was diagnosed with ADEM and received a five-day course of high dose IV steroids, followed by plasmapheresis and second round of high dose IV steroids due to worsening clinical course. Her neurologic exam slowly improved with intensive therapies and prolonged oral steroid taper resulting in almost complete recovery. Three years later, she presented with left hemiparesis and blurry vision. Her repeat MRI showed multiple new periventricular, juxtacortical lesions, and some lesions showed mild enhancement. MRI of the orbits and the spine showed no abnormalities. CSF showed 2 nucleated cells, normal protein, no oligoclonal bands, and elevated myelin basic protein. She was ultimately diagnosed with multiple sclerosis.

### 4.2. Epidemiology and Pathophysiology

Acute disseminated encephalomyelitis (ADEM) is a demyelinating disorder of the CNS. Pediatric ADEM has an incidence of 0.1–0.6/100,000 children [79,80,81,82]. The average age of onset for pediatric ADEM is four to seven years old [53,81,83]. Although variable, there tends to be a slight male predominance [53,81,83]. A majority of cases are preceded by a systemic febrile illness [53,81,84,85]. Case reports of vaccination preceding ADEM also exist, but epidemiological studies found no increased risk of ADEM following vaccines [86,87,88]. The precise etiology of ADEM is unknown. However, a preceding infection, along with other unknown triggers, is thought to initiate a pro-inflammatory state mediated by both humoral and cellular processes in susceptible individuals. Molecular mimicry to self-antigens like myelin basic protein (MBP), proteolipid protein (PLP), and myelin oligodendrocyte glycoprotein (MOG) is theorized to be involved in triggering this inflammatory cascade perhaps by activating complement and natural killer cell-mediated death [71,89,90].

### 4.3. Clinical Presentation

Initial presentation with ADEM is highly variable. Many patients experience a non-specific prodrome of fever, headache, and nausea, which can be mistaken as infection [53,81,85]. Encephalopathy is a requirement for the diagnosis of ADEM [91,92]. Other common neurological symptoms include ataxia and other cerebellar signs (36–47%), pyramidal signs (18–60%), cranial nerve deficits (18–39%), and speech disturbances (7–44%) [53,67,84,89,93,94]. ADEM presentations, by definition, should show signs of polyfocal CNS involvement [91,92].

### 4.4. Diagnosis

The International Pediatric Multiple Sclerosis Society Group (IPMSSG) published consensus definitions for demyelinating disorders of childhood, including ADEM [91,92]. The current consensus definition for ADEM requires the following: (1) A first polyfocal, clinical CNS event with presumed inflammatory demyelinating cause, (2) encephalopathy that cannot be explained by fever, (3) no new clinical or MRI findings emerging three months or more after onset, (4) brain MRI is abnormal during acute (three-month) phase with diffuse, poorly demarcated, large (>1–2 cm) lesions involving predominantly the cerebral white matter, deep gray matter lesions (thalamus or basal ganglia) can be present, but T1 hypointense lesions in the white matter are rare [91,92].

The presence of an infectious prodrome, encephalopathy, and other neurological symptoms should result in the initial consideration of ADEM. Importantly, ADEM is a diagnosis of exclusion necessitating a search for alternative etiologies. The differential diagnosis of ADEM is wide and includes such entities as infectious meningoencephalitis, multiple sclerosis, NMO spectrum disorder, leukodystrophies, CNS vasculitis, CNS malignancies, and neurosarcoidosis, among others. Patients typically undergo blood testing, CSF analysis, neuroimaging, and sometimes EEGs during the acute illness to assist with arriving at the correct diagnosis.

There is no specific blood test for ADEM. Testing for other antibodies associated with acute demyelinating syndromes, such as serum MOG-ab and AQP4-ab, is important to define ADEM, to determine prognosis, and to rule out related disorders. Patients with ADEM who are positive for MOG-ab would meet criteria for MOG-ab-associated disorder [95]. MOG-ab are positive in 33–66% of all pediatric ADEM cases [54,59,67,68,70,96,97,98], and are associated with the development of a non-MS relapsing demyelinating disease course [70]. MOG-ab are positive in 96% of pediatric ADEM cases that develop non-MS recurrent demyelination [70]. Patients presenting with ADEM-like illness who have optic neuritis or transverse myelitis should be screened for AQP4-ab. If positive, they meet diagnostic criteria for NMO-SD [25]. This is relatively uncommon, as almost all patients with ADEM are negative for AQP4-ab [67,70,79,96,98,99], and <10% of AQP4-ab positive NMO-SD cases present with ADEM [79,100]. The incidence of AQP4-ab-negative NMO after ADEM appears to be slightly more common, reported between 2 and 8%, with all affected patients positive for MOG-ab [70,98]. CSF analysis, though non-specific for ADEM, can provide diagnostic support. Lymphocytic pleocytosis is seen in 29–85% of ADEM cases, values typically do not exceed 100 cells/μL [53,93,94,97]. Elevated protein is seen in about 25% of cases [53,93]. Intrathecal oligoclonal bands are rarely seen in ADEM and are only present in 0–20% of cases, again as opposed to MS where OCB are present in the majority of cases [93,94]. Although elevations of pro-inflammatory cytokines/chemokines are seen in ADEM, these tests are not yet used in clinical practice [97]. MRI findings are an essential part of the diagnosis of ADEM. The IPMSSG criteria describes the presence of brain MRI characteristics typical of ADEM including diffuse, poorly demarcated, large lesions involving mostly cerebral white matter. Deep gray matter lesions (thalamus or basal ganglia) may be present, but T1 hypointense lesions in the white matter are rare [91]. Routine EEG can be abnormal showing diffuse slowing or focal spikes [93].

### 4.5. Treatment and Prognosis

There are no large, prospective, randomized, clinical trials of experimental therapeutics in pediatric ADEM, so treatment is based off of case reports, clinical series, and expert opinions. High dose IV glucocorticoids are the most common acute treatment approach for pediatric ADEM. These are usually given as a three to five-day course of IV methylprednisolone or IV dexamethasone followed by an oral steroid taper over four to six weeks [84,101,102,103]. Alternatives to steroids include Intravenous immunoglobulin (IVIG) or plasma exchange [104,105,106,107,108,109,110,111]. In general, it is a common practice among demyelination experts that a repeat examination and MRI be performed after the initial event as well as follow-up for several years to exclude a multiphasic disorder which would warrant further diagnostic workup and a different therapeutic approach.

The prognosis of pediatric ADEM can be assessed in two major domains: (1) clinical recovery outcomes and (2) recurrent demyelination outcomes. Clinical recovery after ADEM has not been uniformly defined. In general, the literature tends to categorize patients into complete recovery or partial recovery (mild to moderate or moderate to severe deficits). Complete recovery is variously defined as EDSS of zero, no reported deficits on a standardized questionnaire at follow-up, normalization of neurologic exam, or lack of any reported deficits by telephone or in written outpatient follow-up records. This is seen in about 50–90% of patients. Mild to moderate deficits (EDSS 1, or persistent mild deficit) are reported with an incidence of 6–65% while moderate to severe deficits (EDSS > 1.5, or persistent moderate to severe deficits) are reported with an incidence of 0–18% [67,85,93]. Studies with a lower percentage of complete recovery and higher percentages of mild to moderate deficits were often looking for more subtle changes utilizing formal neurocognitive testing, underscoring that many of the persistent deficits in ADEM may be underreported or under-recognized. The majority of patients with ADEM will have a monophasic disease with no recurrences after average follow-up periods of at least two years and up to 12 years [53,67,93,112,113]. Multiphasic ADEM, diagnosed following the onset of a second demyelinating event, is seen in up to 12% of pediatric ADEM cases [53,67,93,112,113].

## 5. Metabolic and Mitochondrial Disorders

### 5.1. Clinical Case

**Figure 5:**
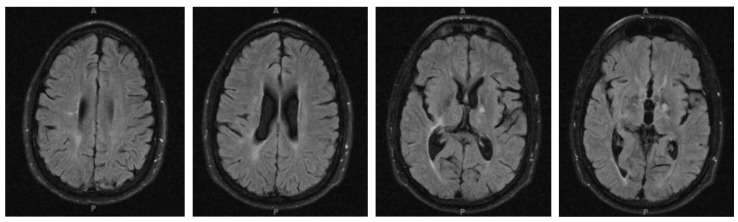
Brain MRI of patient at age 27 (13 years after initial presentation), demonstrating global mild–moderate atrophy and mild periventricular T2/FLAIR signal abnormality, scattered foci of T2 FLAIR hyperintense signal within the bilateral centrum semiovale and basal ganglia. Lacunar infarcts within the left basal ganglia and right thalamus are noted.

A fourteen-year-old boy with a history of arthralgias, fatigue, and frequent headaches presented with four days of right-sided weakness, headache, nausea, vertigo, and difficulty walking following an upper respiratory infection. His exam was notable for all right-sided facial and extremity weakness, hyperreflexia, ataxia, dysmetria, as well as a wide-based gait. A brain MRI showed multiple T2 hyperintense lesions with an acute infarct in the left pons as well as old infarcts in the right pons, bilateral internal capsule, and the right corpus callosum. Differential diagnosis at the time was thought to include vasculitis, a prothrombotic state, cardiac thromboembolism, demyelination, or metabolic disorder. Of note, there was a concern for genetic thrombophilia given the history of stroke at a young age in his mother. He underwent lumbar puncture with CSF showing normal glucose and protein with six nucleated cells. A hypercoagulability workup was sent, which was largely unremarkable except for slightly elevated anti-beta 2 glycoprotein. He was started on aspirin and clopidogrel. Fabry testing was sent off and showed a deficiency of alpha-galactosidase and a confirmed Fabry gene mutation. No similar mutation was identified in his family members. He was subsequently referred to and enrolled in NIH protocol for a clinical trial of Replagal enzyme replacement therapy in children ages 7 to 17 years with Fabry’s disease. With bi-monthly infusions, he had marked improvement in his abdominal pain and headaches, as well as dissipation of his arthralgias. He was eventually transitioned to Fabryzyme. He did go on to have additional recurrent strokes, both clinical and silent (Figure 5).

### 5.2. Epidemiology and Pathophysiology

Inborn errors of metabolism (IEM) are a group of disorders where there is a deficiency of enzyme or transporter activity in a particular metabolic pathway. Inborn errors of metabolism have an incidence and prevalence that differs by specific disease as well as location. The overall incidence of inborn errors of metabolism is 1:1000, but individually, each disease is relatively rare. Rates of IEM tend to correlate with the frequency of consanguinity. Twenty-five percent of patients present with symptoms in the neonatal period, typically after being healthy at birth [114].

Fabry disease, a reported mimic of multiple sclerosis (MS), is an X-linked inherited lysosomal storage disease due to a GLA-gene defect. Fabry disease has a similar gender ratio to MS of 3:1 female: male. A case series in 2013 from Germany found that 67% of females less than 30 years old in the study were likely to present with isolated cerebral findings as their manifestation of the disease, suggesting that women may present with a monosymptomatic course [115].

Mitochondrial disease should also be considered in the differential for MS. For example, mitochondrial encephalomyopathy, lactic acidosis, and stroke-like episodes (MELAS) syndrome is a mitochondrial disorder that leads to multi-organ system dysfunction and is maternally inherited. The reported prevalence differs by location but is considered to be 0.2:100,000 people in Japan and 16–18:100,000 people in Finland. Typically, the age of onset is prior to age 20.5 with 8% of patients presenting before the age of two [116].

### 5.3. Clinical Presentation

The clinical presentation for inborn errors of metabolism differs by disease, but typically all patients are initially symptom-free at birth and then develop symptoms in the first few hours or weeks of life. The time of presentation is based on the affected enzyme. The presentation of inborn errors of metabolism is, therefore, much earlier than pediatric multiple sclerosis, in general.

One exception to early clinical presentation is Fabry disease in which there are multiple reported cases of patients misdiagnosed with multiple sclerosis. In Fabry disease, the GLA gene defect leads to glycosphingolipid deposition in multiple organ systems which manifests as cardiomyopathy, angiokeratomas, vasculopathy, renal dysfunction, cornea verticillata, deafness, visual disturbances, and progressive white matter lesions. With variable symptoms, Fabry disease is challenging to diagnose and the mean delay from symptom onset to diagnosis is 10–16 years. Neurologic symptoms appear earliest with paresthesias, neuropathic pain, and white matter lesions in the cerebrum. The vasculopathy affects small and large vessels. Symptoms may occur as relapsing or remitting sensory deficits due to microangiopathic alterations [115].

With the relapsing-remitting symptoms and white matter lesions, there are cases of Fabry disease fulfilling McDonald criteria for multiple sclerosis. Similar to MS, Fabry disease may be monosymptomatic in its involvement of CNS rather than presenting with classical symptoms, such as angiokeratoma, renal or cardiac disease. Patients may present with gait disturbances, double vision, or ataxia. One female patient in the case series aforementioned, presented with visual disturbances at age 14. She was diagnosed at age 27 with MS and then later diagnosed instead with Fabry disease at 28 [115].

MELAS presents with neurologic manifestations including seizure, myopathy, cortical blindness, headaches, and dementia, in addition to stroke-like episodes. 84 to 99% percent of individuals with MELAS develop stroke-like episodes. These episodes present as acute and often transient symptoms which may include altered mental status, hemiparesis, hemianopsia, or aphasia [117].

### 5.4. Diagnosis

Early diagnosis is paramount in IEM as early therapy must be done expeditiously to prevent death. Initial laboratory studies should be sent if an IEM is being considered including plasma, urine, and cerebrospinal fluid studies [114]. Molecular genetic testing and enzyme studies can help to confirm. These studies are all valuable in making a diagnosis. Tandem mass spectroscopy has been utilized specifically for screening measures in the newborn period [114].

In Fabry disease, there are features on diagnostic workup that can help to differentiate it from MS. On MRI, progressive white matter lesions are seen at early stages of Fabry disease, often before clinical symptoms. These progressive white matter lesions are often disseminated with punctuate and subcortical white matter changes [115]. The typical MS lesions are located on the corpus callosum with “Dawson fingers” which are not seen in Fabry patients. Fabry patients also have normal spinal imaging. The “pulvinar sign” is another differentiating brain MRI finding historically associated with Fabry disease. This is a hyperintensity of the lateral pulvinar nucleus that is noted on a T1 weighted image and has been previously regarded as pathognomonic for Fabry disease. It is thought to be related to the accumulation of globotriaosylceramide. This sign has since been noted to be associated with other metabolic disorders, including Creutzfeldt–Jakob or Tay–Sachs disease [118]. A recent retrospective study in 2017, analyzed the brain imaging of 133 patients with Fabry disease and found that only four out of 133 (3.0%) subjects had a positive “pulvinar sign [118].” All four of the patients who had pulvinar findings were men in renal failure and under enzyme replacement therapy. For males in the study, this represented 7.5% of the male population. With these new study results, the pulvinar sign is no longer being considered as sensitive for Fabry disease [118]. CSF abnormalities are seen in Fabry disease typically, including pleocytosis and elevated protein levels [115]. Oligoclonal bands are a non-specific finding but sensitive in MS. Fabry disease should be especially considered in a female patient if they present with asymmetric white matter lesions (without corpus callosum involvement), have no spinal MRI involvement, lack oligoclonal bands in CSF, have renal or cardiac manifestations, or have a family history of death at an early age from cardiac or renal disease [115].

In MELAS, neuroimaging obtained during acute stroke-like episodes shows focal lesions often located in the parietal or occipital lobes, but these lesions do not follow the typical vascular distribution areas. Uniquely, MR spectroscopy shows lactate accumulation with a decrease in N-acetylaspartate, whereas MR angiography is normal. The level of lactate on MR spectroscopy directly relates to the level of neurologic impairment. Unlike MS, MELAS presents with increased lactate and pyruvate in CSF [116].

### 5.5. Treatment and Prognosis

Treatment and prognosis vary amongst specific forms of inborn errors of metabolism. The prognosis for Fabry disease improves with early enzyme replacement therapy and leads to improved quality of life due to symptom reduction. If therapy is delayed, patients may develop organ damage that is irreversible. It is, therefore, crucial to differentiate between Fabry disease and MS early in the symptomatic course.

The majority of therapies for MELAS are supportive. L-arginine has been shown in the acute phase to be beneficial in the recovery of stroke-like symptoms [116].

## 6. Leukodystrophies

### 6.1. Clinical Case

**Figure 6:**
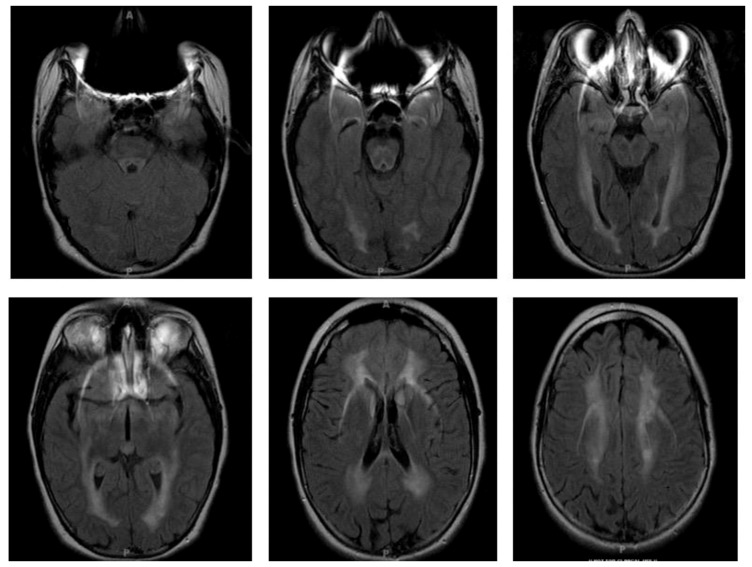
Brain MRI of patient at age 13 (four years after initial presentation), demonstrating global moderate atrophy and leukomalacic changes of the cerebral white matter and deep portion of the cerebellum.

A seventeen-year-old girl was referred to our clinic with progressively worsening weakness and ataxia over several years. Her initial symptoms started at nine years of age with dysphagia and choking, and her MRI showed a FLAIR hyperintense lesion in the pons, which led to the diagnosis of brainstem glioma without biopsy given the depth of the lesion. She received radiation therapy at an outside hospital followed by six weeks of steroid treatment, which led to improvement initially. After that, she developed progressive weakness, ataxia and became wheelchair bound over the next few years. By the time we saw her in clinic, she had received multiple brain MRI’s (Figure 6), the latter of which showed global moderate atrophy and leukomalacic changes of the cerebral white matter and deep portion of the cerebellum. There was substantial atrophy of the brainstem, particularly in the medulla. At this consult, a leukodystrophy was suspected, and the diagnosis of juvenile Alexander disease was confirmed through GFAP mutation testing.

### 6.2. Epidemiology and Pathophysiology

Leukodystrophies are genetic white matter disorders due to inherited myelin defects which affect the central nervous system with or without peripheral involvement. Typically, symptoms are progressive. There is limited data on the overall frequency of leukodystrophies. Retrospective data from Germany’s national health reporting system suggests an incidence of 1 in 50,000 live births present with genetic white matter disorders. In the United States, data in Utah showed an incidence of 1 in 8000 live births. This large difference may be due to distinct patient population differences in the compared locations as well as the differences in defining a leukodystrophy [119].

Leukodystrophies vary in their disease mechanism with mutations in genes specific for components within the white matter, such as axons, astrocytes, and microglia, as well as myelin or oligodendrocytes. There are also cases where the pathophysiology is still unknown. A new classification system for leukodystrophies was suggested by Van der Knaap and Bugiani in 2017. The past classification system categorized leukodystrophies into myelin disorders including demyelinating, hypomyelinating, or myelin vacuolization disorders. With greater discoveries of the pathophysiology of leukodystrophies, there are many leukodystrophies which do not fit into the categories of myelin disorders. The new classification system also includes astrocytopathies, microgliopathies, axonopathies, and vasculopathies of white matter [120]. Most leukodystrophies are autosomal recessive [120].

### 6.3. Clinical Presentation

Leukodystrophies are heterogeneous in their presentations and prognosis. In most cases, patients present with the nonspecific finding of loss of motor function, which is also a common presenting symptom in acquired demyelinating diseases. This could be seen in children who are considered to be clumsy and fall frequently or in infants who are developmentally delayed in reaching motor milestones. Patients may present with central weakness first as the corticospinal tracts may be involved early in the disease course. Spasticity, chorea, dystonia, or other movement disorders may be seen if upper motor neuron pathways and deep gray nuclei are involved [121]. With cerebellar involvement, progressive ataxia is seen. Peripheral involvement is less common, but it can occur in the form of a sensory neuropathy. This can cause mixed ataxia in combination with the cerebellar involvement. Other neurologic manifestations include seizure, personality change, autonomic dysfunction, and worsening school performance [121].

Certain specific leukodystrophies can be recognized based off of unique clinical features in addition to the loss of motor function or neurologic manifestations, as well as specific physical exam findings. For instance, Alexander disease presents with recurrent vomiting, and palatal myoclonus is a typical exam finding. [121].

Clinical presentation often occurs in the first few years of life, such as in Alexander disease, but can occur in adolescence or adulthood. Unlike those with MS, patients with leukodystrophies rarely have a relapsing and remitting course and instead have a more progressive and chronic clinical course. Relapses have been noted in cases of vanishing white matter disease and metachromatic leukodystrophy after episodes of trauma [122].

### 6.4. Diagnosis

The diagnosis of leukodystrophies relies on taking a careful personal history and family history, focusing on members with movement disorders, seizures, or cognitive delays, as well as parental consanguinity. Physical examination is vital as unique physical features are associated with specific leukodystrophies. Once there is suspicion for a leukodystrophy, confirmatory testing includes biochemical studies like an arylsulfatase A activity assay for metachromatic leukodystrophy, as well as genetic testing (single gene, multi-gene panels, or whole exome sequencing). Neuroimaging, specifically brain MRI, is an important aspect of identifying leukodystrophies as there are diagnostic patterns seen when combining the imaging with the clinical picture. For example, lesions with dissemination in space and time would be unlikely in leukodystrophies and more consistent with MS. However, there are rare leukodystrophies which have associated multifocal MRI findings which can mimic that of MS. Early in the clinical course of leukodystrophies, there is a higher risk of misdiagnosing with MS. The timing of the MRI can be crucial as MRI scans obtained within the first year of life prove difficult to analyze hypomyelination versus demyelination. Therefore, serial MRI scans are recommended. Due to this risk of misdiagnosis, one must recognize the diagnostic findings which help differentiate between MS and leukodystrophies. For instance, leukodystrophies present with symmetric involvement of peripheral nerves and spinal tracts on neurophysiologic studies that measure visual-evoked potentials. This is in contrast to the prolonged asymmetric latencies that are seen with MS. Findings suggestive of a leukodystrophy include a family history concerning for genetic disorder, atypical MRI findings for MS, and CSF that lacks oligoclonal bands [122].

There are three distinguishing characteristics on MRI that help to diagnose a leukodystrophy. The first is the presence of hypomyelination with the level of involvement determined by the sequence obtained. For instance, shortening is seen with myelination on T1 before T2. Therefore, T1 is crucial for diagnostic interpretation of the imaging. T1-weighted signal is hypointense for demyelinating leukodystrophies but hypointense or isointense for hypomyelinated disorders. T2 weighted hyperintensity must be seen for diagnosis of a leukodystrophy [123]. The second characteristic is whether the abnormalities on imaging are confluent or isolated and multifocal [124]. Multifocal and isolated changes are often more consistent with acquired disorders like an infection. On the other hand, confluent and bilateral white matter abnormalities are seen with leukodystrophies. If the findings are confluent, the third distinguishing characteristic is the location of the abnormalities. The lesion locations vary amongst leukodystrophies. For example, Alexander disease has a frontal pattern, whereas metachromatic leukodystrophy is periventricular. Spinal MRI should also be performed to determine if there is spine involvement, as this would be less suggestive of a leukodystrophy. CT of the brain is beneficial for detecting calcifications [121].

Early differentiation of white matter disorders is crucial, especially to prevent unnecessary tests from being performed on patients with acquired white matter abnormalities. This promotes more expedited treatment for acquired disorders, including MS.

### 6.5. Treatment and Prognosis

As opposed to the multiple therapeutic options available to modify disease course in MS, there are rarely primary treatments for leukodystrophies due to diverse and often unknown mechanisms of disease. The goal is to perform symptomatic management and comfort care. A multidisciplinary approach is necessary [123].

Primary manifestations of the disease can rarely be treated, but bone marrow transplant or hematopoietic stem cell transplant may be beneficial in Krabbe disease, X-linked adrenoleukodystrophy, and metachromatic leukodystrophy. Transplantation can halt the progression of symptoms [125]. Novel approaches, including gene therapy and gene editing, are currently being researched [126].

Prognosis differs by leukodystrophy, but those leukodystrophies which present with progressive symptoms are often fatal. Alexander disease is untreatable and fatal within 10 years of symptom onset. Genetic counseling is recommended with most leukodystrophies following an autosomal recessive inheritance.

## 7. Conclusions

As exemplified by the clinical cases discussed in this review, diagnosing pediatric MS and specifically distinguishing this condition from other inflammatory and non-inflammatory mimics continues to be a challenge in clinical practice. The development of consensus criteria for MS as well as other demyelinating entities, such as NMOSD and ADEM, has undoubtedly contributed in the delineation of these diseases, but there continues to be an unquestionable degree of overlap in clinical and MRI features [25,53,91]. Similarly, MOG-ab associated disorders now represent a disease category which may have pathophysiological and phenotypical features that distinguish them from MS and NMOSD and large prospective studies are in urgent need to understand it [77]. Moreover, an erroneous diagnosis of MS in non-inflammatory etiologies, such as metabolic disorders and leukodystrophies, can be made even while applying established criteria. Therefore, more complete diagnostic workups are needed to arrive at the correct diagnosis in some scenarios [115,122]. Continuing to learn not only about the features that distinguish these differential diagnoses but also how best to treat each of them through collaborative research, will ultimately facilitate the optimization of care for pediatric demyelinating diseases.

## References

[1] Chitnis T., Ness J., Krupp L., Waubant E., Hunt T., Olsen C.S., Rodriguez M., Lotze T., Gorman M., Benson L. (2016). Clinical features of neuromyelitis optica in children: US Network of Pediatric MS Centers report. Neurology.

[2] Fragoso Y.D., Adoni T., Bichuetti D.B., Brooks J.B., Ferreira M.L., Oliveira E.M., Oliveira C.L., Ribeiro S.B., Silva A.E., Siquineli F. (2013). Neuromyelitis optica and pregnancy. J. Neurol..

[3] Bourre B., Marignier R., Zephir H., Papeix C., Brassat D., Castelnovo G., Collongues N., Vukusic S., Labauge P., Outteryck O. (2012). Neuromyelitis optica and pregnancy. Neurology.

[4] Confavreux C., Hutchinson M., Hours M.M., Cortinovis-Tourniaire P., Moreau T. (1998). Rate of pregnancy-related relapse in multiple sclerosis. Pregnancy in Multiple Sclerosis Group. N. Engl. J. Med..

[5] Kim S.H., Mealy M.A., Levy M., Schmidt F., Ruprecht K., Paul F., Ringelstein M., Aktas O., Hartung H.P., Asgari N. (2018). Racial differences in neuromyelitis optica spectrum disorder. Neurology.

[6] Lennon V.A., Wingerchuk D.M., Kryzer T.J., Pittock S.J., Lucchinetti C.F., Fujihara K., Nakashima I., Weinshenker B.G. (2004). A serum autoantibody marker of neuromyelitis optica: Distinction from multiple sclerosis. Lancet.

[7] Lennon V.A., Kryzer T.J., Pittock S.J., Verkman A.S., Hinson S.R. (2005). IgG marker of optic-spinal multiple sclerosis binds to the aquaporin-4 water channel. J. Exp. Med..

[8] Takahashi T., Fujihara K., Nakashima I., Misu T., Miyazawa I., Nakamura M., Watanabe S., Shiga Y., Kanaoka C., Fujimori J. (2007). Anti-aquaporin-4 antibody is involved in the pathogenesis of NMO: A study on antibody titre. Brain.

[9] Lucchinetti C.F., Guo Y., Popescu B.F., Fujihara K., Itoyama Y., Misu T. (2014). The pathology of an autoimmune astrocytopathy: Lessons learned from neuromyelitis optica. Brain Pathol..

[10] Takano R., Misu T., Takahashi T., Sato S., Fujihara K., Itoyama Y. (2010). Astrocytic damage is far more severe than demyelination in NMO: A clinical CSF biomarker study. Neurology.

[11] Weinshenker B.G., Wingerchuk D.M. (2017). Neuromyelitis Spectrum Disorders. Mayo Clin. Proc..

[12] Keegan M., Konig F., McClelland R., Bruck W., Morales Y., Bitsch A., Panitch H., Lassmann H., Weinshenker B., Rodriguez M. (2005). Relation between humoral pathological changes in multiple sclerosis and response to therapeutic plasma exchange. Lancet.

[13] Palace J., Leite M.I., Nairne A., Vincent A. (2010). Interferon Beta treatment in neuromyelitis optica: Increase in relapses and aquaporin 4 antibody titers. Arch. Neurol..

[14] Gelfand J.M., Cotter J., Klingman J., Huang E.J., Cree B.A. (2014). Massive CNS monocytic infiltration at autopsy in an alemtuzumab-treated patient with NMO. Neurol. Neuroimmunol. Neuroinflamm..

[15] Min J.H., Kim B.J., Lee K.H. (2012). Development of extensive brain lesions following fingolimod (FTY720) treatment in a patient with neuromyelitis optica spectrum disorder. Mult. Scler..

[16] Kleiter I., Hellwig K., Berthele A., Kumpfel T., Linker R.A., Hartung H.P., Paul F., Aktas O. (2012). Failure of natalizumab to prevent relapses in neuromyelitis optica. Arch. Neurol..

[17] Jarius S., Wildemann B., Paul F. (2014). Neuromyelitis optica: Clinical features, immunopathogenesis and treatment. Clin. Exp. Immunol..

[18] Brum D.G., Barreira A.A., dos Santos A.C., Kaimen-Maciel D.R., Matiello M., Costa R.M., Deghaide N.H., Costa L.S., Louzada-Junior P., Diniz P.R. (2010). HLA-DRB association in neuromyelitis optica is different from that observed in multiple sclerosis. Mult. Scler..

[19] Cree B.A., Spencer C.M., Varrin-Doyer M., Baranzini S.E., Zamvil S.S. (2016). Gut microbiome analysis in neuromyelitis optica reveals overabundance of Clostridium perfringens. Ann. Neurol..

[20] Varrin-Doyer M., Spencer C.M., Schulze-Topphoff U., Nelson P.A., Stroud R.M., Cree B.A., Zamvil S.S. (2012). Aquaporin 4-specific T cells in neuromyelitis optica exhibit a Th17 bias and recognize Clostridium ABC transporter. Ann. Neurol..

[21] Wingerchuk D.M., Lennon V.A., Pittock S.J., Lucchinetti C.F., Weinshenker B.G. (2006). Revised diagnostic criteria for neuromyelitis optica. Neurology.

[22] Devic E. (1894). Myélite subaiguë compliquée de névrite optique. Le Bulletin Médicale.

[23] Popescu B.F., Lennon V.A., Parisi J.E., Howe C.L., Weigand S.D., Cabrera-Gomez J.A., Newell K., Mandler R.N., Pittock S.J., Weinshenker B.G. (2011). Neuromyelitis optica unique area postrema lesions: Nausea, vomiting, and pathogenic implications. Neurology.

[24] McKeon A., Lennon V.A., Lotze T., Tenenbaum S., Ness J.M., Rensel M., Kuntz N.L., Fryer J.P., Homburger H., Hunter J. (2008). CNS aquaporin-4 autoimmunity in children. Neurology.

[25] Wingerchuk D.M., Banwell B., Bennett J.L., Cabre P., Carroll W., Chitnis T., de Seze J., Fujihara K., Greenberg B., Jacob A. (2015). International consensus diagnostic criteria for neuromyelitis optica spectrum disorders. Neurology.

[26] Jarius S., Ruprecht K., Wildemann B., Kuempfel T., Ringelstein M., Geis C., Kleiter I., Kleinschnitz C., Berthele A., Brettschneider J. (2012). Contrasting disease patterns in seropositive and seronegative neuromyelitis optica: A multicentre study of 175 patients. J. Neuroinflamm..

[27] Banwell B., Tenembaum S., Lennon V.A., Ursell E., Kennedy J., Bar-Or A., Weinshenker B.G., Lucchinetti C.F., Pittock S.J. (2008). Neuromyelitis optica-IgG in childhood inflammatory demyelinating CNS disorders. Neurology.

[28] Bradshaw M.J., Vu N., Hunley T.E., Chitnis T. (2017). Child Neurology: Neuromyelitis optica spectrum disorders. Neurology.

[29] Jarius S., Franciotta D., Paul F., Ruprecht K., Bergamaschi R., Rommer P.S., Reuss R., Probst C., Kristoferitsch W., Wandinger K.P. (2010). Cerebrospinal fluid antibodies to aquaporin-4 in neuromyelitis optica and related disorders: Frequency, origin, and diagnostic relevance. J. Neuroinflamm..

[30] Poser C.M., Paty D.W., Scheinberg L., McDonald W.I., Davis F.A., Ebers G.C., Johnson K.P., Sibley W.A., Silberberg D.H., Tourtellotte W.W. (1983). New diagnostic criteria for multiple sclerosis: Guidelines for research protocols. Ann. Neurol..

[31] Jarius S., Paul F., Franciotta D., Ruprecht K., Ringelstein M., Bergamaschi R., Rommer P., Kleiter I., Stich O., Reuss R. (2011). Cerebrospinal fluid findings in aquaporin-4 antibody positive neuromyelitis optica: Results from 211 lumbar punctures. J. Neurol. Sci..

[32] Klawiter E.C., Alvarez E., Xu J., Paciorkowski A.R., Zhu L., Parks B.J., Cross A.H., Naismith R.T. (2009). NMO-IgG detected in CSF in seronegative neuromyelitis optica. Neurology.

[33] McKeon A., Pittock S.J., Lennon V.A. (2011). CSF complements serum for evaluating paraneoplastic antibodies and NMO-IgG. Neurology.

[34] Kira J. (2011). Neuromyelitis optica and opticospinal multiple sclerosis: Mechanisms and pathogenesis. Pathophysiology.

[35] Kurtzke J.F. (1983). Rating neurologic impairment in multiple sclerosis: An expanded disability status scale (EDSS). Neurology.

[36] Warabi Y., Matsumoto Y., Hayashi H. (2007). Interferon beta-1b exacerbates multiple sclerosis with severe optic nerve and spinal cord demyelination. J. Neurol. Sci..

[37] Wingerchuk D.M., Pittock S.J., Lucchinetti C.F., Lennon V.A., Weinshenker B.G. (2007). A secondary progressive clinical course is uncommon in neuromyelitis optica. Neurology.

[38] Kessler R.A., Mealy M.A., Levy M. (2016). Treatment of Neuromyelitis Optica Spectrum Disorder: Acute, Preventive, and Symptomatic. Curr. Treat. Options Neurol..

[39] Abboud H., Petrak A., Mealy M., Sasidharan S., Siddique L., Levy M. (2016). Treatment of acute relapses in neuromyelitis optica: Steroids alone versus steroids plus plasma exchange. Mult. Scler..

[40] Kleiter I., Gahlen A., Borisow N., Fischer K., Wernecke K.D., Wegner B., Hellwig K., Pache F., Ruprecht K., Havla J. (2016). Neuromyelitis optica: Evaluation of 871 attacks and 1,153 treatment courses. Ann. Neurol..

[41] Cree B.A., Lamb S., Morgan K., Chen A., Waubant E., Genain C. (2005). An open label study of the effects of rituximab in neuromyelitis optica. Neurology.

[42] Nosadini M., Alper G., Riney C.J., Benson L.A., Mohammad S.S., Ramanathan S., Nolan M., Appleton R., Leventer R.J., Deiva K. (2016). Rituximab monitoring and redosing in pediatric neuromyelitis optica spectrum disorder. Neurol. Neuroimmunol. Neuroinflamm..

[43] Costanzi C., Matiello M., Lucchinetti C.F., Weinshenker B.G., Pittock S.J., Mandrekar J., Thapa P., McKeon A. (2011). Azathioprine: Tolerability, efficacy, and predictors of benefit in neuromyelitis optica. Neurology.

[44] Jacob A., Matiello M., Weinshenker B.G., Wingerchuk D.M., Lucchinetti C., Shuster E., Carter J., Keegan B.M., Kantarci O.H., Pittock S.J. (2009). Treatment of neuromyelitis optica with mycophenolate mofetil: Retrospective analysis of 24 patients. Arch. Neurol..

[45] Pittock S.J., Berthele A., Fujihara K., Kim H.J., Levy M., Palace J., Nakashima I., Terzi M., Totolyan N., Viswanathan S. (2019). Eculizumab in Aquaporin-4-Positive Neuromyelitis Optica Spectrum Disorder. N. Engl. J. Med..

[46] Collongues N., Marignier R., Zephir H., Papeix C., Fontaine B., Blanc F., Rodriguez D., Fleury M., Vukusic S., Pelletier J. (2010). Long-term follow-up of neuromyelitis optica with a pediatric onset. Neurology.

[47] Collongues N., Marignier R., Jacob A., Leite M.I., Siva A., Paul F., Zephir H., Akman-Demir G., Elsone L., Jarius S. (2014). Characterization of neuromyelitis optica and neuromyelitis optica spectrum disorder patients with a late onset. Mult. Scler..

[48] Kang H., Chen T., Li H., Xu Q., Cao S., Wei S. (2017). Prognostic factors and disease course in aquaporin-4 antibody-positive Chinese patients with acute optic neuritis. J. Neurol..

[49] Sellner J., Boggild M., Clanet M., Hintzen R.Q., Illes Z., Montalban X., Du Pasquier R.A., Polman C.H., Sorensen P.S., Hemmer B. (2010). EFNS guidelines on diagnosis and management of neuromyelitis optica. Eur. J. Neurol..

[50] Kim S.H., Jeong I.H., Hyun J.W., Joung A., Jo H.J., Hwang S.H., Yun S., Joo J., Kim H.J. (2015). Treatment Outcomes with Rituximab in 100 Patients With Neuromyelitis Optica: Influence of FCGR3A Polymorphisms on the Therapeutic Response to Rituximab. JAMA Neurol..

[51] Tradtrantip L., Zhang H., Saadoun S., Phuan P.W., Lam C., Papadopoulos M.C., Bennett J.L., Verkman A.S. (2012). Anti-aquaporin-4 monoclonal antibody blocker therapy for neuromyelitis optica. Ann. Neurol..

[52] Papadopoulos M.C., Verkman A.S. (2012). Aquaporin 4 and neuromyelitis optica. Lancet Neurol..

[53] Boesen M.S., Blinkenberg M., Koch-Henriksen N., Thygesen L.C., Uldall P.V., Magyari M., Born A.P. (2018). Implications of the International Paediatric Multiple Sclerosis Study Group consensus criteria for paediatric acute disseminated encephalomyelitis: A nationwide validation study. Dev. Med. Child Neurol..

[54] Hacohen Y., Absoud M., Deiva K., Hemingway C., Nytrova P., Woodhall M., Palace J., Wassmer E., Tardieu M., Vincent A. (2015). Myelin oligodendrocyte glycoprotein antibodies are associated with a non-MS course in children. Neurol. Neuroimmunol. Neuroinflamm..

[55] Reindl M., Di Pauli F., Rostasy K., Berger T. (2013). The spectrum of MOG autoantibody-associated demyelinating diseases. Nat. Rev. Neurol..

[56] Rostasy K., Mader S., Schanda K., Huppke P., Gartner J., Kraus V., Karenfort M., Tibussek D., Blaschek A., Bajer-Kornek B. (2012). Anti-myelin oligodendrocyte glycoprotein antibodies in pediatric patients with optic neuritis. Arch. Neurol..

[57] Tenembaum S., Chitnis T., Nakashima I., Collongues N., McKeon A., Levy M., Rostasy K. (2016). Neuromyelitis optica spectrum disorders in children and adolescents. Neurology.

[58] Fernandez-Carbonell C., Vargas-Lowy D., Musallam A., Healy B., McLaughlin K., Wucherpfennig K.W., Chitnis T. (2016). Clinical and MRI phenotype of children with MOG antibodies. Mult. Scler..

[59] Ketelslegers I.A., Van Pelt D.E., Bryde S., Neuteboom R.F., Catsman-Berrevoets C.E., Hamann D., Hintzen R.Q. (2015). Anti-MOG antibodies plead against MS diagnosis in an Acquired Demyelinating Syndromes cohort. Mult. Scler..

[60] Hacohen Y., Wong Y.Y., Lechner C., Jurynczyk M., Wright S., Konuskan B., Kalser J., Poulat A.L., Maurey H., Ganelin-Cohen E. (2018). Disease Course and Treatment Responses in Children with Relapsing Myelin Oligodendrocyte Glycoprotein Antibody-Associated Disease. JAMA Neurol..

[61] Brunner C., Lassmann H., Waehneldt T.V., Matthieu J.M., Linington C. (1989). Differential ultrastructural localization of myelin basic protein, myelin/oligodendroglial glycoprotein, and 2’,3’-cyclic nucleotide 3’-phosphodiesterase in the CNS of adult rats. J. Neurochem..

[62] Johns T.G., Bernard C.C. (1999). The structure and function of myelin oligodendrocyte glycoprotein. J. Neurochem..

[63] Lebar R., Lubetzki C., Vincent C., Lombrail P., Boutry J.M. (1986). The M2 autoantigen of central nervous system myelin, a glycoprotein present in oligodendrocyte membrane. Clin. Exp. Immunol..

[64] Linington C., Lassmann H. (1987). Antibody responses in chronic relapsing experimental allergic encephalomyelitis: Correlation of serum demyelinating activity with antibody titre to the myelin/oligodendrocyte glycoprotein (MOG). J. Neuroimmunol..

[65] Peschl P., Bradl M., Hoftberger R., Berger T., Reindl M. (2017). Myelin Oligodendrocyte Glycoprotein: Deciphering a Target in Inflammatory Demyelinating Diseases. Front. Immunol..

[66] Lalive P.H., Hausler M.G., Maurey H., Mikaeloff Y., Tardieu M., Wiendl H., Schroeter M., Hartung H.P., Kieseier B.C., Menge T. (2011). Highly reactive anti-myelin oligodendrocyte glycoprotein antibodies differentiate demyelinating diseases from viral encephalitis in children. Mult. Scler..

[67] Baumann M., Sahin K., Lechner C., Hennes E.M., Schanda K., Mader S., Karenfort M., Selch C., Hausler M., Eisenkolbl A. (2015). Clinical and neuroradiological differences of paediatric acute disseminating encephalomyelitis with and without antibodies to the myelin oligodendrocyte glycoprotein. J. Neurol. Neurosurg Psychiatr..

[68] Probstel A.K., Dornmair K., Bittner R., Sperl P., Jenne D., Magalhaes S., Villalobos A., Breithaupt C., Weissert R., Jacob U. (2011). Antibodies to MOG are transient in childhood acute disseminated encephalomyelitis. Neurology.

[69] Rostasy K., Mader S., Hennes E.M., Schanda K., Gredler V., Guenther A., Blaschek A., Korenke C., Pritsch M., Pohl D. (2013). Persisting myelin oligodendrocyte glycoprotein antibodies in aquaporin-4 antibody negative pediatric neuromyelitis optica. Mult. Scler..

[70] Hennes E.M., Baumann M., Schanda K., Anlar B., Bajer-Kornek B., Blaschek A., Brantner-Inthaler S., Diepold K., Eisenkölbl A., Gotwald T. (2017). Prognostic relevance of MOG antibodies in children with an acquired demyelinating syndrome. Neurology.

[71] Brilot F., Dale R.C., Selter R.C., Grummel V., Kalluri S.R., Aslam M., Busch V., Zhou D., Cepok S., Hemmer B. (2009). Antibodies to native myelin oligodendrocyte glycoprotein in children with inflammatory demyelinating central nervous system disease. Ann. Neurol..

[72] Thulasirajah S., Pohl D., Davila-Acosta J., Venkateswaran S. (2016). Myelin Oligodendrocyte Glycoprotein-Associated Pediatric Central Nervous System Demyelination: Clinical Course, Neuroimaging Findings, and Response to Therapy. Neuropediatrics.

[73] Pohl D., Rostasy K., Reiber H., Hanefeld F. (2004). CSF characteristics in early-onset multiple sclerosis. Neurology.

[74] Dale R.C., Tantsis E.M., Merheb V., Kumaran R.Y., Sinmaz N., Pathmanandavel K., Ramanathan S., Booth D.R., Wienholt L.A., Prelog K. (2014). Antibodies to MOG have a demyelination phenotype and affect oligodendrocyte cytoskeleton. Neurol. Neuroimmunol. Neuroinflamm..

[75] Rostasy K., Reindl M. (2013). Role of autoantibodies in acquired inflammatory demyelinating diseases of the central nervous system in children. Neuropediatrics.

[76] von Budingen H.C., Hauser S.L., Ouallet J.C., Tanuma N., Menge T., Genain C.P. (2004). Frontline: Epitope recognition on the myelin/oligodendrocyte glycoprotein differentially influences disease phenotype and antibody effector functions in autoimmune demyelination. Eur. J. Immunol..

[77] Dos Passos G.R., Oliveira L.M., da Costa B.K., Apostolos-Pereira S.L., Callegaro D., Fujihara K., Sato D.K. (2018). MOG-IgG-Associated Optic Neuritis, Encephalitis, and Myelitis: Lessons Learned from Neuromyelitis Optica Spectrum Disorder. Front. Neurol..

[78] Baumann M., Hennes E.M., Schanda K., Karenfort M., Kornek B., Seidl R., Diepold K., Lauffer H., Marquardt I., Strautmanis J. (2016). Children with multiphasic disseminated encephalomyelitis and antibodies to the myelin oligodendrocyte glycoprotein (MOG): Extending the spectrum of MOG antibody positive diseases. Mult. Scler..

[79] Absoud M., Lim M.J., Chong W.K., De Goede C.G., Foster K., Gunny R., Hemingway C., Jardine P.E., Kneen R., Likeman M. (2013). Paediatric acquired demyelinating syndromes: Incidence, clinical and magnetic resonance imaging features. Mult. Scler..

[80] Pohl D., Hennemuth I., von Kries R., Hanefeld F. (2007). Paediatric multiple sclerosis and acute disseminated encephalomyelitis in Germany: Results of a nationwide survey. Eur. J. Pediatr..

[81] Torisu H., Kira R., Ishizaki Y., Sanefuji M., Yamaguchi Y., Yasumoto S., Murakami Y., Shimono M., Nagamitsu S., Masuzaki M. (2010). Clinical study of childhood acute disseminated encephalomyelitis, multiple sclerosis, and acute transverse myelitis in Fukuoka Prefecture, Japan. Brain Dev..

[82] Xiong C.H., Yan Y., Liao Z., Peng S.H., Wen H.R., Zhang Y.X., Chen S.H., Li J., Chen H.Y., Feng X.W. (2014). Epidemiological characteristics of acute disseminated encephalomyelitis in Nanchang, China: A retrospective study. BMC Public Health.

[83] Langer-Gould A., Zhang J.L., Chung J., Yeung Y., Waubant E., Yao J. (2011). Incidence of acquired CNS demyelinating syndromes in a multiethnic cohort of children. Neurology.

[84] Dale R.C., de Sousa C., Chong W.K., Cox T.C., Harding B., Neville B.G. (2000). Acute disseminated encephalomyelitis, multiphasic disseminated encephalomyelitis and multiple sclerosis in children. Brain.

[85] Erol I., Ozkale Y., Alkan O., Alehan F. (2013). Acute disseminated encephalomyelitis in children and adolescents: A single center experience. Pediatr. Neurol..

[86] Hviid A., Svanstrom H., Scheller N.M., Gronlund O., Pasternak B., Arnheim-Dahlstrom L. (2018). Human papillomavirus vaccination of adult women and risk of autoimmune and neurological diseases. J. Intern. Med..

[87] Langer-Gould A., Qian L., Tartof S.Y., Brara S.M., Jacobsen S.J., Beaber B.E., Sy L.S., Chao C., Hechter R., Tseng H.F. (2014). Vaccines and the risk of multiple sclerosis and other central nervous system demyelinating diseases. JAMA Neurol..

[88] Scheller N.M., Svanstrom H., Pasternak B., Arnheim-Dahlstrom L., Sundstrom K., Fink K., Hviid A. (2015). Quadrivalent HPV vaccination and risk of multiple sclerosis and other demyelinating diseases of the central nervous system. JAMA.

[89] Fujinami R.S., Oldstone M.B. (1985). Amino acid homology between the encephalitogenic site of myelin basic protein and virus: Mechanism for autoimmunity. Science.

[90] Mader S., Gredler V., Schanda K., Rostasy K., Dujmovic I., Pfaller K., Lutterotti A., Jarius S., Di Pauli F., Kuenz B. (2011). Complement activating antibodies to myelin oligodendrocyte glycoprotein in neuromyelitis optica and related disorders. J. Neuroinflamm..

[91] Krupp L.B., Tardieu M., Amato M.P., Banwell B., Chitnis T., Dale R.C., Ghezzi A., Hintzen R., Kornberg A., Pohl D. (2013). International Pediatric Multiple Sclerosis Study Group criteria for pediatric multiple sclerosis and immune-mediated central nervous system demyelinating disorders: Revisions to the 2007 definitions. Mult. Scler..

[92] Krupp L.B., Banwell B., Tenembaum S. (2007). Consensus definitions proposed for pediatric multiple sclerosis and related disorders. Neurology.

[93] Pavone P., Pettoello-Mantovano M., Le Pira A., Giardino I., Pulvirenti A., Giugno R., Parano E., Polizzi A., Distefano A., Ferro A. (2010). Acute disseminated encephalomyelitis: A long-term prospective study and meta-analysis. Neuropediatrics.

[94] Yamaguchi Y., Torisu H., Kira R., Ishizaki Y., Sakai Y., Sanefuji M., Ichiyama T., Oka A., Kishi T., Kimura S. (2016). A nationwide survey of pediatric acquired demyelinating syndromes in Japan. Neurology.

[95] Lopez-Chiriboga A.S., Majed M., Fryer J., Dubey D., McKeon A., Flanagan E.P., Jitprapaikulsan J., Kothapalli N., Tillema J.M., Chen J. (2018). Association of MOG-IgG Serostatus With Relapse After Acute Disseminated Encephalomyelitis and Proposed Diagnostic Criteria for MOG-IgG-Associated Disorders. JAMA Neurol..

[96] de Mol C.L., Wong Y.Y.M., van Pelt E.D., Ketelslegers I.A., Bakker D.P., Boon M., Braun K.P.J., van Dijk K.G.J., Eikelenboom M.J., Engelen M. (2018). Incidence and outcome of acquired demyelinating syndromes in Dutch children: Update of a nationwide and prospective study. J. Neurol..

[97] Kothur K., Wienholt L., Mohammad S.S., Tantsis E.M., Pillai S., Britton P.N., Jones C.A., Angiti R.R., Barnes E.H., Schlub T. (2016). Utility of CSF Cytokine/Chemokines as Markers of Active Intrathecal Inflammation: Comparison of Demyelinating, Anti-NMDAR and Enteroviral Encephalitis. PLoS ONE.

[98] Duignan S., Wright S., Rossor T., Cazabon J., Gilmour K., Ciccarelli O., Wassmer E., Lim M., Hemingway C., Hacohen Y. (2018). Myelin oligodendrocyte glycoprotein and aquaporin-4 antibodies are highly specific in children with acquired demyelinating syndromes. Dev. Med. Child Neurol..

[99] Huppke P., Rostasy K., Karenfort M., Huppke B., Seidl R., Leiz S., Reindl M., Gartner J. (2013). Acute disseminated encephalomyelitis followed by recurrent or monophasic optic neuritis in pediatric patients. Mult. Scler..

[100] Kim S.M., Waters P., Woodhall M., Yang J.W., Yang H., Kim J.E., Sung J.J., Park K.S., Lee K.W. (2014). Characterization of the spectrum of Korean inflammatory demyelinating diseases according to the diagnostic criteria and AQP4-Ab status. BMC Neurol..

[101] Hynson J.L., Kornberg A.J., Coleman L.T., Shield L., Harvey A.S., Kean M.J. (2001). Clinical and neuroradiologic features of acute disseminated encephalomyelitis in children. Neurology.

[102] Tenembaum S., Chamoles N., Fejerman N. (2002). Acute disseminated encephalomyelitis: A long-term follow-up study of 84 pediatric patients. Neurology.

[103] Anlar B., Basaran C., Kose G., Guven A., Haspolat S., Yakut A., Serdaroglu A., Senbil N., Tan H., Karaagaoglu E. (2003). Acute disseminated encephalomyelitis in children: Outcome and prognosis. Neuropediatrics.

[104] Kleiman M., Brunquell P. (1995). Acute disseminated encephalomyelitis: Response to intravenous immunoglobulin. J. Child Neurol..

[105] Pradhan S., Gupta R.P., Shashank S., Pandey N. (1999). Intravenous immunoglobulin therapy in acute disseminated encephalomyelitis. J. Neurol. Sci..

[106] Sahlas D.J., Miller S.P., Guerin M., Veilleux M., Francis G. (2000). Treatment of acute disseminated encephalomyelitis with intravenous immunoglobulin. Neurology.

[107] Nishikawa M., Ichiyama T., Hayashi T., Ouchi K., Furukawa S. (1999). Intravenous immunoglobulin therapy in acute disseminated encephalomyelitis. Pediatr. Neurol..

[108] Stricker R.B., Miller R.G., Kiprov D.D. (1992). Role of plasmapheresis in acute disseminated (postinfectious) encephalomyelitis. J. Clin. Apher..

[109] Balestri P., Grosso S., Acquaviva A., Bernini M. (2000). Plasmapheresis in a child affected by acute disseminated encephalomyelitis. Brain Dev..

[110] Miyazawa R., Hikima A., Takano Y., Arakawa H., Tomomasa T., Morikawa A. (2001). Plasmapheresis in fulminant acute disseminated encephalomyelitis. Brain Dev..

[111] Khurana D.S., Melvin J.J., Kothare S.V., Valencia I., Hardison H.H., Yum S., Faerber E.N., Legido A. (2005). Acute disseminated encephalomyelitis in children: Discordant neurologic and neuroimaging abnormalities and response to plasmapheresis. Pediatrics.

[112] Suppiej A., Cainelli E., Casara G., Cappellari A., Nosadini M., Sartori S. (2014). Long-term neurocognitive outcome and quality of life in pediatric acute disseminated encephalomyelitis. Pediatr. Neurol..

[113] Neuteboom R.F., Boon M., Catsman Berrevoets C.E., Vles J.S., Gooskens R.H., Stroink H., Vermeulen R.J., Rotteveel J.J., Ketelslegers I.A., Peeters E. (2008). Prognostic factors after a first attack of inflammatory CNS demyelination in children. Neurology.

[114] El-Hattab A.W. (2015). Inborn errors of metabolism. Clin. Perinatol..

[115] Bottcher T., Rolfs A., Tanislav C., Bitsch A., Kohler W., Gaedeke J., Giese A.K., Kolodny E.H., Duning T. (2013). Fabry disease—Underestimated in the differential diagnosis of multiple sclerosis?. PLoS ONE.

[116] El-Hattab A.W., Adesina A.M., Jones J., Scaglia F. (2015). MELAS syndrome: Clinical manifestations, pathogenesis, and treatment options. Mol. Genet. Metab..

[117] Ito H., Mori K., Kagami S. (2011). Neuroimaging of stroke-like episodes in MELAS. Brain Dev..

[118] Cocozza S., Russo C., Pisani A., Olivo G., Riccio E., Cervo A., Pontillo G., Feriozzi S., Veroux M., Battaglia Y. (2017). Redefining the Pulvinar Sign in Fabry Disease. AJNR Am. J. Neuroradiol..

[119] Vanderver A., Hussey H., Schmidt J.L., Pastor W., Hoffman H.J. (2012). Relative incidence of inherited white matter disorders in childhood to acquired pediatric demyelinating disorders. Semin. Pediatr. Neurol..

[120] van der Knaap M.S., Bugiani M. (2017). Leukodystrophies: A proposed classification system based on pathological changes and pathogenetic mechanisms. Acta Neuropathol..

[121] Parikh S., Bernard G., Leventer R.J., van der Knaap M.S., van Hove J., Pizzino A., McNeill N.H., Helman G., Simons C., Schmidt J.L. (2015). A clinical approach to the diagnosis of patients with leukodystrophies and genetic leukoencephelopathies. Mol. Genet. Metab..

[122] Kohler W. (2008). Diagnostic algorithm for the differentiation of leukodystrophies in early MS. J. Neurol..

[123] Vanderver A., Prust M., Tonduti D., Mochel F., Hussey H.M., Helman G., Garbern J., Eichler F., Labauge P., Aubourg P. (2015). Case definition and classification of leukodystrophies and leukoencephalopathies. Mol. Genet. Metab..

[124] Schiffmann R., van der Knaap M.S. (2009). Invited article: An MRI-based approach to the diagnosis of white matter disorders. Neurology.

[125] Eichler F., Grodd W., Grant E., Sessa M., Biffi A., Bley A., Kohlschuetter A., Loes D.J., Kraegeloh-Mann I. (2009). Metachromatic leukodystrophy: A scoring system for brain MR imaging observations. AJNR Am. J. Neuroradiol..

[126] Gordon-Lipkin E., Fatemi A. (2018). Current Therapeutic Approaches in Leukodystrophies: A Review. J. Child Neurol..

